# Frailty Status Typologies in Spanish Older Population: Associations with Successful Aging

**DOI:** 10.3390/ijerph17186772

**Published:** 2020-09-17

**Authors:** José M. Tomás, Trinidad Sentandreu-Mañó, Irene Fernández

**Affiliations:** 1 Department of Methodology for the Behavioural Sciences, University of Valencia, 46010 Valencia, Spain; Jose.M.Tomas@uv.es (J.M.T.); Irene.Fernandez@uv.es (I.F.); 2 Advanced Research Methods Applied to Quality of Life Promotion (ARMAQoL), University of Valencia, 46010 Valencia, Spain; 3 Department of Physiotherapy, University of Valencia, 46010 Valencia, Spain

**Keywords:** older adults, frailty profiles, latent class analysis, quality of life, perceived health

## Abstract

Background: Defining frailty typologies would contribute to guiding specific care interventions. These typologies could additionally be related to different health outcomes. This study aims at identifying subgroups of frail older adults based on the physical frailty phenotype and examining the relationships of these frailty profiles with quality of life and perceived health. Methods: This study relies on data from the SHARE project, namely a representative sample of 1765 Spanish-dwelling older adults identified as frail or pre-frail. Analysis included general descriptive statistics, exploratory latent class analysis (LCA) to determine the number of frailty subgroups, and LCA with covariates to examine differential relationships with markers of successful aging. Results: Statistical criteria and interpretability of the classes suggested that the LCA model with four classes should be retained. Class 1 was identified as the “frail people” group, Class 2 “activity problems” group, Class 3 “fatigued” group, and those belonging to Class 4 “lack of strength” group. Final LCA with covariates showed lower levels of quality of life and perceived health of the “frail” as compared to other frailty subgroups. Conclusion: This study revealed four different patterns of frailty attributes and further offered evidence on individuals’ differential status of health regarding distinct frailty conditions.

## 1. Introduction

The aging of society constitutes an important challenge for health care systems due to the increase in the life expectancy [1]. The current generation of older adults expects to age well, and to maintain their general well-being and, ultimately, enhance the quality of later life [2].

A fundamental issue in elderly care is targeting those older people at risk and in need of care interventions [3]. An age-related condition is frailty, which is a syndrome probably due to multiple causes and characterized by diminished strength, endurance, and physiological function that promotes dependency and ultimately death [4]. Different operational definitions of frailty have been proposed, but the most commonly used is the frailty phenotype by Fried et al. [5]. Although there is no consensus, this definition has been used as a gold-standard in many studies [6]. Criteria describing Fried’s phenotype include: unintentional weight loss, exhaustion, weakness, slowness and reduced physical activity. In this regard, other studies have used modified versions [7,8,9] or other physical frailty criteria [10] in order to define this syndrome.

Further specification of frailty by defining profiles of frail older people contributes to the ongoing debate on the conceptualization of frailty and could improve interventions [3]. Evidence suggests that frail individuals are not a clinically homogeneous group [11], but to date, the heterogeneity in the frail population has not been fully acknowledged in care interventions [3]. Identification of different profiles within the frailty people may be of great help in clinical settings, in order to better manage frail people [12].

Some studies have looked at profiles or clusters of frailty. Some of them have used cluster analytical techniques, while others have used latent class analytic techniques. All these studies have had different scopes, for example using samples from the general population [13,14,15], only a part of the general population such as women [16], or patients of a hospital [17]. Additionally, the studies on frailty profiles have used a variety of indicators to get the profiles. Some of these studies only used Fried’s criteria [13,14,15,16,18], while others included psychosocial indicators of frailty and/or related problems [3,11,17,19].

Few studies have looked for classes or clusters only employing the physical dimensions of frailty. Among them, Bandeen-Roche et al. [16] studied older women (65 years or older) in the general population and used latent class analysis (LCA) to get classes from the five physical conditions of the frailty syndrome. Their results point out two classes, frail and robust older women. Frail women had a higher risk of disability, institutionalization, or death. Chen et al. [13] also studied a sample of community-dwelling older adults, men and women, and performed LCA analyses on the five physical indicators of frailty, and again found two classes, frail and robust. Then, they used logistic regression to relate the two classes with other variables and found frailty associated with age, poorer health, more depression and anxiety, less social activity, not consuming alcohol and higher rates of cognitive impairment. Lohman et al. [15] also employed LCA on the five indicators of frailty in a sample of 51 years or older Americans, and found the same two classes (frail and robust) and frailty condition was associated to a number of negative health outcomes. Nevertheless, they also estimated LCA including persistent pain and they argued that the classes from this LCA model better related to health outcomes. However, Liu et al. [14] employed LCA to find frailty classes in a sample of community-dwelling Taiwanese adults aged 50 or older, and contrary to the aforementioned evidence, they found four classes: robust, mobility group, low activity and non-mobility group.

With regard to the outcomes frailty may be linked to, quality of life (QoL) is a salient one in old age. The concept of QoL includes dimensions such as the feeling of well-being and the health-related quality of life (HRQoL), which are strong indicators of successful aging [20]. Different studies have shown a negative association between physical frailty and quality of life [21,22,23,24,25,26] or perceived health [9,21,23,27,28,29,30,31], but there is no evidence whatsoever on how quality of life may be differently related to different frailty profiles. However, relating frailty profiles to quality of life and healthy aging markers allows for a patient-centered approach rather than an approach centered in the syndrome.

Therefore, the aim of the present study was: firstly, to identify subgroups or profiles of frailty in older adults based on the physical conditions that define the frailty status; and secondly, to estimate the relationships of these profiles with quality of life and perceived health indicators. The novelty of this research is analyzing only pre-frail and frail subjects in order to avoid the simple clustering into two groups of frail and non-frail. That is, the aim of this research is to distinguish classes within the people that are already frail or at least have pre-frail conditions.

## 2. Materials and Methods

### 2.1. Sample and Procedure

This study was carried out using data from the Survey of Health, Aging and Retirement in Europe (SHARE) Wave 6 [32,33]. SHARE is a longitudinal study focused on the study of European populations aged 50 and older. Data were gathered using probability-based sampling, whose further details can be found in Malter and Börsch-Supan [34].

From the 6th Wave of SHARE data, we selected the pre-frail and frail Spanish-dwelling participants that were 60 years old and older, yielding a total of 1765 individuals.

### 2.2. Instruments and Measures

Frailty was measured as previously operationalized in SHARE [7,8]. This frailty approach is based on the five criteria established by Fried et al. [5] and has been tested and validated by different authors [8,35,36]. The specific five attributes used in this study were:

- Unintentional weight loss was operationalized using the question “What has your appetite been like”. It scored positive when the participants reported “a diminution in desire for food”. In the case of an uninterpretable response to the question, the participant was asked whether they had been eating more or less than usual. Answering “less” was also considered a positive indicator of unintentional weight loss.
- Fatigue, resulting from a positive response to the question, “In the last month, have you had too little energy to do things you wanted to do?”
- Slowness was defined as a positive answer to any of the following two mobility questions strongly associated with low speed: “Because of a health problem, do you have difficulty walking 100 m?’ or “Because of a health problem, do you have difficulty climbing one flight of stairs without resting?”. Both questions referred to difficulties lasting more than three months.
- Weakness was assessed by handgrip strength measurements (twice for each hand) using a dynamometer. The maximum grip strength measure was analyzed according to the cut-off points stratified by gender and body max index, as proposed by Fried et al. [5].
- Physical activity was measured using the question “How often do you engage in activities that require a moderate level of energy such as gardening, cleaning the car, or going for a walk?” The criterion was fulfilled for participants answering either “one to three times a month” or “hardly ever or never.”

One point was allocated for each fulfilled criterion. Participants with zero points were classified as robust, those with one or two points were classified as pre-frail, and those with three to five points were classified as frail [5]. Robust older adults were excluded from the study.

Quality of life was measured using the abridged version of the Control, Autonomy, Self-realization, Pleasure scale (CASP-19) [37] designed for SHARE purposes [38]. Items were answered in a 4-point Likert scale ranging from 1 (never) to 4 (often). A total score was obtained by summing all item scores. The final score ranged from 12 to 48, with the highest values indicating better quality of life.

Perceived health was measured by means of an item of general perceived health included within the SF-36 [39], namely “Would you say your health is…?”. A 5-point Likert scale was used, ranging from 1 (poor) to 5 (excellent).

### 2.3. Ethical Clearance

The Ethical Approval for gathering of the data used in this study was obtained by the SHARE project and it can be publicly consulted at: http://www.share-project.org/fileadmin/pdf_documentation/MPG_Ethics_Council_SHARE_overall_approval_29.05.2020__en_.pdf. More information at: http://www.share-project.org/.

### 2.4. Statistical Analyses

Descriptive statistics for all variables under study were calculated in SPSS 26. Mplus 8 [40] was used for latent mixture modeling. All models were estimated with robust (full information) maximum likelihood estimation (MLR). The mixture model used was LCA. In LCA, subgroup membership is not observed and must be inferred from the data [41]. LCA was used in an exploratory way, and the number of classes retained was based on several statistical criteria. Firstly, we used information criteria such as the Bayesian information criterion (BIC), sample size-adjusted BIC (ABIC), and Akaike information criterion (AIC), with smaller values indicating better fit. Secondly, entropy, a statistic that assesses accuracy and can range from 0 to 1 (perfect accuracy), was considered. Statistical model comparison likelihood ratio tests and bootstrapping procedures were also used—pecifically, the Lo-Mendell-Rubin test (LMR) [42] and the Bootstrap likelihood ratio test (BLRT) [43]. These tests compare the improvement between neighboring class models with a statistically significant result interpreted as fit improvement due to the extra class. Beyond these criteria, interpretability of the results was also considered [44]. The recent developments in LCA consider relating the indicators to the latent classes and also relating the classes extracted to a set of external variables [45]. Once the number of latent classes was determined, groups of participants based on these classes were compared based on several markers of successful aging. This new LCA with covariates was also tested. In this LCA with covariates, quality of life and perceived health were treated as continuous.

## 3. Results

### 3.1. Descriptive Statistics

Among the 1765 participants, 1044 participants out of the total sample were female (59.2%) and the remaining 721 were male (40.8%). Their mean age was 75.22 years old (SD = 8.86). Overall, 1285 (72.8%) were pre-fail and 480 (27.2%) were frail, classified according to the Fried frailty phenotype [5]. Descriptive statistics of the categorical variables involved in the study are shown in Table 1. Prefrail and frail groups were compared by age and gender. There was a significant mean difference in age (t (1763) = −7.17, *p* < 0.001), with frail people being older (Mean = 77.66, SD = 8.5) than pre-frail people (Mean = 74.31, SD = 8.82). Regarding gender, there was also a significant association with frailty condition (χ^2^(1) = 14.57, *p* < 0.001). Among the women, 30.6% were frail, while only 22.3% of the men were.

### 3.2. Frailty Classes

LCAs from one to four classes were estimated, because with only five frailty indicators more than four classes do not reduce complexity. The model with one class was used as a baseline model against which to compare the models with extra classes. Table 2 shows all statistical criteria considered to decide the number of classes retained. The model with four classes had the lowest information criteria, and had statistically significant LMR and BLRT tests. However, the best entropy data were obtained with three classes. Therefore, the criteria are slightly contradictory, and attending to results by Nylund et al. [46], we have given priority to the results of the BRLT test and BIC because they work better for this type of model. We also found four classes being more interpretable than three.

### 3.3. Relations with the Latent Classes

Once the number of classes to retain has been decided, we proceed to estimate an LCA with four classes and four covariates, age, gender as control variables, and health status and quality of life as markers of quality of life. Model fit statistics for this model were even better than those of the LCA with four classes and no covariates. Model fit was: AIC = 7998.5, BIC = 8184.1, ABIC = 8072.8, with entropy = 0.755. Class 1 included 526 (35.51%) participants, with class 2 including 207 or 13.98% of the cases, class 3 had 364 (24.58%) cases, and the rest (384, 25.92%) of the participants were included in class 4.

Table 3 offers the conditional probabilities of each class for every indicator of frailty. These conditional probabilities allow for interpreting the sub-groups or classes. Class 1 has relatively high probabilities in all frailty indicators, and thus this group represents “frail people”. Class 2 is characterized for high probabilities in slowness and activity, this group represents people with “activity problems”. Class 3 has very high probability of being fatigued and very low probabilities in the rest of indicators, and therefore are old adults that are “fatigued”. Finally, class 4 is characterized by being high in the weakness indicator (lack of strength) and therefore will be labeled “lack of strength”.

**Figure 1:**
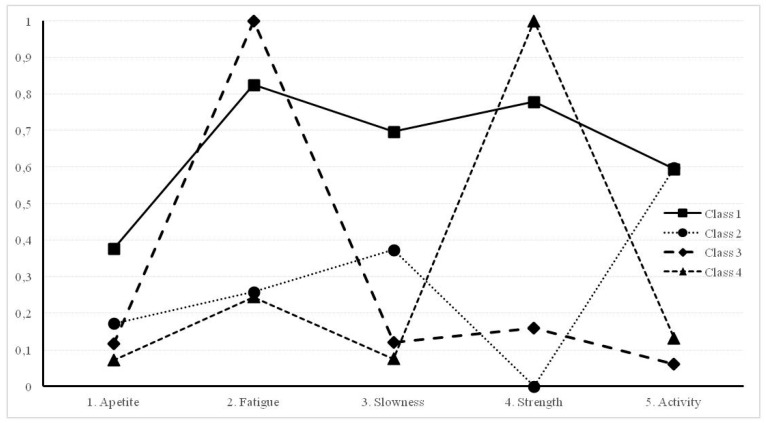
Conditional probabilities of symptoms depending on the class.

A graphical representation of the conditional probabilities is offered in Figure 1.

In this LCA model with covariates, the key point is the effects of the covariates on the classes. Class 1 (frail people) is taken as the reference group, and therefore all effects in a class are compared to frail people. The effects in terms of coefficients and odds-ratios are presented in Table 4. All effects of age, gender, quality of life and perceived health were statistically significant.

Regarding the effects of covariates in the comparison between class 2 (mobility problems) and class 1 (frail), the sign of the coefficients indicates that an increase in age make a person more likely to be frail than having only mobility problems, and the same is true for being male. However, increases in quality of life and perceived health increase the probability of being in the group of mobility group rather than in the frailty group. When odds ratios are considered, the probability of being in the frailty group and not in the mobility problems group is 1.14-fold increased per year of age, while it is increased 1.62-fold if you are a man. On the contrary, the odds of being in the mobility group (vs. frail) increases 5.88-fold with each point increase in perceived health.

When class 3 (fatigued) is compared to class 1 (frail), it follows the same pattern of relationship. That is, being older and male makes a person more likely to be in the frail group, while a better quality of life and health increase the likelihood of being in the fatigued group (vs. frail). In terms of odd-ratios, being a year older increases the odds of being frail (vs. fatigued) 1.24-fold. Being male increases the odds of being frail (vs. fatigued) 1.70-fold. Regarding quality of life and health, we estimated that the odds of being in the fatigued group (vs. frail) increases 1.18-fold with a change of one point in quality of life and 5.34-fold per one unit change in perceived health.

Finally, class 4 (lack of strength) is compared with class 1 (frail), and again the pattern of relationships remains the same. The odds of being frail (vs. lack of strength) increases 1.08-fold with each added year of life, and 3.03-fold for being male. On the other hand, the odds of being in the group of lack of strength (vs. frail) increases 1.26 with each unit increase in quality of life, and 7.011 with each unit increase in perceived.

In sum, the analyses of the effects reflect that age and being male are associated with the probabilities of being frail vs. the other “less severe” groups of frailty symptoms. On the contrary, a better perceived health and quality of life are expected in classes 2 (mobility problems), 3 (fatigued), and 4 (lack of strength) compared to the frail group.

## 4. Discussion

The present study reports four different classes of frailty. All individuals involved in the study were already displaying pre-frail or frail conditions, implying that the four subtypes of frailty are all substantive. The “Frail” class (35.51% of the sample) is composed of those individuals with high probabilities of displaying all five indicators of physical frailty. The “Activity problems” class (13.98%) comprises older adults who most likely present slowness and physical inactivity. The “fatigued” class (24.58%) is made up of individuals whose only expected symptom is fatigue, and who do not have problems regarding any other indicators of physical frailty. Finally, “lack of strength” class (25.92%) is similar to the “fatigued” class in that it includes individuals whose only probable ailment is a relevant lack of strength, but who are expected to perform well in any other indicator.

One of the goals of the study was to disentangle frailty subtypes only among those individuals whose frail or pre-frail physical conditions were already evident. Previous studies on general population samples found a two-class solution of frail vs. non-frail individuals [13,14,15,16]. An exception was the study by Liu et al. [14], in which up to four subtypes of frailty were found. In the four-class model retained by the authors, more than half of the sample was identified as non-frail, and the rest were assigned to three different groups: mobility-type frailty, with a higher prevalence of slowness and weakness; non-mobility-type frailty, with higher exhaustion and weight loss; and the low physical activity group. To some point, the mobility-type frailty was similar to the “activity problems” class found in our study, while non-mobility-type frailty is more similar to the “fatigued” class. However, in the study by Liu et al. [14], weight loss and fatigue were not salient symptoms for any of the four classes, nor was there a frail class. Although the physical indicators may be associated through interrelated pathways, different underlying mechanisms may determine these subtypes. Wasting or chronic inflammatory processes may contribute to the non-mobility group, and, on the other hand, the mobility group could be largely due to neurodegeneration [14]. Existing bibliography refers to the existence of different trigger conditions that could activate the frailty process [47,48]. Initially, such conditions could be associated with the physical manifestations of frailty that characterize different frailty profiles obtained in this study: “fatigued”, “activity problems” and “lack of strength”. Studies show that alterations of the neuroendocrine system, micronutrients deficiency, or other factors related to energy and nutritional imbalance could be key point in this activation of frailty [47]. These alterations could be related to fatigue, among other manifestations. The literature has also referred to a lack of activity as a precursor of frailty and how sedentary lifestyle can be decisive in this process [47,49]. On the other hand, Fried et al. [5] presented a construct based on energy imbalance and sarcopenia to activate the cycle of frailty, whose first manifestation could be weakness [48].

A second goal of this study was relating frailty profiles to successful aging-related outcomes. Indicators of successful aging employed in the study were quality of life and perceived health. We compared all classes against the “frail” class, in order to determine whether the effects of frailty on quality of life and perceived health varied between those with high probabilities of displaying all physical indicators of frailty and those whose frailty conditions were not as widespread. The results show statistically significant better quality of life and perceived health in “activity problems”, “fatigued” and “lack of strength” classes compared to the “frail” class. These results are in line with previous literature pointing to a negative association between quality of life and physical frailty, and between perceived health and physical frailty [9,21,22,23,24,25,26,27,28,30,31]. Some of the studies classified the general population and obtained two groups that then related to outcomes such as health and wellbeing indicators, which makes it difficult to compare their results with ours because our groups only include pre-frail and frail people [13,15,16]. However, their overall results show that frailty is negatively associated with health and wellbeing measures, which is in line with our results in the sense that the frail group with the presence of most indicators of a frail condition had worse health and worse quality of life (wellbeing). Additionally, our results offer a first glimpse of the idiosyncratic relationships between markers of successful aging and frailty profiles. Thus, the “activity problems”, “fatigued” and “lack of strength” frailty typologies had higher probabilities (with odd ratios of 1.22, 1.18 and 1.26, respectively) of displaying better quality of life than the “frail” frailty status typology. On its part, perceived health followed similar patterns of relationship, but the odd ratios were higher in the “lack of strength” typology (odd-ratio 7.01), where a higher score of perceived health is more likely when compared to the “frail” typology, followed by the “mobility problems” typology (5.88) and “fatigued” typology (5.34). This result points out that as the number of frailty symptoms accumulates, the negative impact on quality of life and perceived health is stronger. In other words, the presence of more frailty symptoms in a person notoriously undermines self-reported health and quality of life.

Age and gender were also included as covariates in LCA models to provide a better description of the profiles. A large volume of the literature has demonstrated that frailty is associated with age, when comparing frail versus non-frail groups [5,9,13]. Additionally, the evidence generated in this study shows that increased age increases the odds of being in the “frail” class, described as the most severe one (or at least with higher probabilities in all criteria). Regarding gender differences, several studies have consistently shown a higher prevalence of frailty in women [50]. In line with the literature data, we observed that the proportion of frail women was significantly higher than the proportion of frail men according to the modified version of Fried’s phenotype. Additionally, when the frailty status typologies were considered, this study shows that being male is associated with the probability of being in the frail subtype. This class has high probabilities in the five frailty attributes and therefore could be considered the most severe class compared to the other groups of manifestations. These findings could be masked when using an overall frailty label and could give some explanation to another gender-related condition in the existing bibliography, the higher mortality among men resulting from frailty [18,51,52]. Sex-specific pathways to frailty could also explain specific gender-related profiles [19].

Among studies examining different frailty profiles or clusters, the ones using the physical operationalization of frailty employed samples of the general population in Japan [13], Taiwan [14] and the United States [15], or a sample of the general female population in the United States [16]. As all these studies took place in North America or Asia, there is a lack of knowledge in reference to frailty subtypes or classes in other populations. The present study offered evidence of frailty profiles in a representative sample of Spanish-dwelling older adults, giving a first glimpse of frailty subtypes in Europe. Moreover, in three out of four of these previous studies, the same two frailty classes were found, representing the robust and frail groups of individuals [13,15,16]. By excluding non-frail individuals, this study revealed four different patterns of frailty attributes, which sets the basis for differential treatment in order to lessen the detrimental effects of frailty in quality of life and perceived health.

Despite the contributions made by this study, there are some limitations too. For example, despite examining differences in quality of life and perceived health among classes, there is still no evidence on how these frailty classes may differently predict markers of successful aging, or vice-versa. Previous longitudinal evidence points to a bidirectional relationship between frailty and quality of life [24] and a causal relationship between frailty and reduced health-related quality of life [28]. The cross-sectional design of our study does not allow conclusions of directional relationships between frailty classes and markers of good aging. Moreover, in this study, frailty was operationalized by its physical indicators. However, other operationalizations of frailty, or a combination of them, were also employed in the literature. Even though physical frailty has been used as the gold-standard for frailty measurement [6], these other frailty measurements are also possible and might lead to different results.

## 5. Conclusions

All in all, this study contributes to the pre-existing literature on frailty classes by studying substantive frailty typologies. To the best of our knowledge, this is the first time a representative sample of Spanish-dwelling older adult population has been used, meaning that inferences about population can be drawn and hence “frail”, “activity problems”, “fatigued” and “lack of strength” classes perfectly mirror frailty heterogeneity in Spanish dwelling older adults. This research also examined differences in quality of life and perceived health among “activity problems”, “fatigued” and “lack of strength” classes against the “frail” class. However, future research should focus on interclass comparisons among all classes using these and other age-relevant variables, in order to gather information regarding the specific characteristics of every frailty class, which could foster patient-centered intervention development.

## Figures and Tables

**Table 1:** Descriptive statistics of the categorical variables involved in the study.

Variable	Mean ± SD or n (%)
Gender	
Female	1044 (59.2%)
Male	721 (40.8)
Frailty condition	
Pre-frail	1285 (72.8%)
Frail	480 (27.2%)
Age	75.22 (8.86)
Appetite (Loss)	321 (20.5)
Fatigue (Yes)	1002 (63.9)
Slowness (Yes)	731 (41.4)
Strength (Lack)	767 (57.1)
Activity (Inactive)	728 (41.2)
Perceived Health	
Poor	451 (25.6)
Fair	672 (38.1)
Good	508 (28.8)
Very good	114 (6.5)
Excellent	20 (1.1)
Quality of Life	33.40 ± 6.32

Notes: SD = Standard Deviation; n = number of observations.

**Table 2:** Models’ fit for 1 to 4 classes.

#Classes	AIC	BIC	ABIC	Entropy	LMR Test	*p*	BLR Test	*p*
1	10,271.1	10,298.5	10,282.6	NA	NA	NA	NA	NA
2	9993.4	10,053.7	10,018.7	0.717	283.3	<0.001	289.6	<0.001
3	9828.1	9921.2	9867.2	0.787	173.4	<0.001	177.3	<0.001
4	9737.3	9863.2	9790.2	0.680	100.5	<0.001	102.8	<0.001

Notes: AIC = Akaike information criterion; BIC = Bayesian information criterion; ABIC = adjusted BIC; LMR = Lo-Mendell-Rubin test; BLRT = bootstrapped log-likelihood ratio test; NA = not applicable.

**Table 3:** Conditional probabilities of the manifestations in each class.

Indicators	Class 1. Frail n = 526	Class 2. Mobility Problems n = 207	Class 3. Fatigued n = 364	Class 4. Lack of Strength n = 384
1. Appetite	0.377	0.172	0.117	0.072
2. Fatigue	0.826	0.259	1.00	0.245
3. Slowness	0.697	0.374	0.120	0.076
4. Strength	0.779	0.000	0.160	1.00
5. Activity	0.595	0.598	0.062	0.132

**Table 4:** Effects, standard errors, odds-ratio, confidence intervals and significance tests for all covariates in the model with class 1 as the reference group.

**Covariate**	**Class 2 vs. Class 1**						
	**Effect**	**SE**	***p***	**Odd-Ratio**	**SE**	***95% CI***	***p***
Age	−0.133	0.02	<0.01	0.875	0.02	0.835–0.914	<0.01
Gender (0 = female, 1 = male)	−0.489	0.03	0.11	0.614	0.18	0.254–0.974	0.04
Quality of Life	0.023	0.03	<0.01	1.225	0.04	1.145–1.305	<0.01
Perceived Health	1.772	0.31	<0.01	5.882	1.81	2.262–9.502	<0.01
**Covariate**	**Class 3 vs. Class 1**						
	**Effect**	**SE**	***p***	**Odd-Ratio**	**SE**	***95% CI***	***p***
Age	−0.18	0.02	<0.01	0.804	0.01	0.784–0.824	<0.01
Gender (0 = female, 1 = male)	−0.53	0.28	0.06	0.585	0.16	0.265–0.905	0.01
Quality of Life	0.169	0.03	<0.01	1.184	0.04	1.104–1.264	<0.01
Perceived Health	1.676	0.25	<0.01	5.347	1.37	2.607–8.807	<0.01
**Covariate**	**Class 4 vs. Class 1**						
	**Effect**	**SE**	***p***	**Odd-Ratio**	**SE**	***95% CI***	***p***
Age	−0.084	0.02	<0.01	0.919	0.02	0.879–0.959	<0.01
Gender (0 = female, 1 = male)	−1.099	0.28	<0.01	0.333	0.09	0.153–0.513	<0.01
Quality of Life	0.169	0.03	<0.01	1.265	0.04	1.185–1.345	<0.01
Perceived Health	1.679	0.26	<0.01	7.011	1.86	3.290–10.73	<0.01
